# The nature of “internal sensations” of higher brain functions may be derived from the design rules for artificial machines that can produce them

**DOI:** 10.1186/1754-1611-6-21

**Published:** 2012-11-05

**Authors:** Kunjumon I Vadakkan

**Affiliations:** 1 Division of Neurology, Faculty of Medicine, University of Manitoba, GF532-820 Sherbrook Street, Winnipeg, R3A1R9, MB, Canada

## Abstract

Modeling various neuronal functions in search of emergent properties may achieve success when the gold standard of replicating the models in physical systems starts exhibiting some of these properties. Since very large number of functions can be modeled and need testing, we suggest an alternate method of examining higher brain functions: seeing them as internal sensations formed from their hypothetical basic units. Here, we explain the need to replicate the natural mechanism using electronic circuits, discuss some of the technical aspects and introduce some concepts for searching for properties of internal sensations evolving from them.

## Introduction

Understanding the nature of conscious internal sensations in the brain remains a challenge [1]. The central issue is that when certain products of a system’s operation are available only to the system in the form of internal sensations, a second-person perspective becomes inadequate to study them. Measuring the remaining products of the operation, such as behavioral motor outputs, does not provide information about the properties of internal sensations. A typical example is the difficulty in replicating the transferable mechanism of natural memory needed to develop artificial intelligence (AI). Previous studies [2-5] examined changes taking place at the synaptic junctions between the neurons (the junction between presynaptic (axonal) terminal of one order neuron and the postsynaptic terminal (dendritic spine) of the next order neuron) during the acquisition of information from the environment with the hope to find an explanation for this mechanism. At the systems level, studies have examined the wiring patterns using simple behavioral paradigms, microscopic examinations and genetic dissections of the neuronal circuits [6-8]. Even though all 303 neurons of the worm C.elegans and their connections have been known for the last 25 years [9], an artificial neural network of the same type with the emergent properties that are expected to evolve from its synaptic connections could not be created using current knowledge in the subject. This points to the possibilities for emergent properties evolving from the nervous system that may not have any relationship with the sub-level elements that build the neuronal networks similar to that of a cell’s organizational networks [10] or those elements arising from the collective dynamics of “small world” networks [11].

### What are internal sensations?

Internal sensations such as consciousness and memory can only be accessed by the owner of the nervous system. Even though the virtual nature of internal sensations has been of concern since the time of Descartes, philosophers have maintained the radical view that Cartesian sensations represent something real [12,13]. Internal sensations are virtual sensations characteristic of the remaining properties (or associated learned sensory inputs) of an item or an event that are perceived by the nervous system in the presence of one of the qualities from the item (or a separate cue stimulus that was previously associatively learned). For example, by looking at an equilateral triangle, one may perceive the measurement of the angles as 60 degrees, provided the information was previously associatively learned. Furthermore, a mechanism that can explain subjective qualities (first-person perspective) of conscious experience may be considered a requirement of a system [14] capable of creating internal sensations. This makes higher brain functions different from systems that sense changes at the level of the molecules and cells, such as hepato-portal sensors of hypoglycemia [15], internal coherence seen in embryonic development [16], and self-replication of prion proteins [17].

### Limitations of detecting internal sensations from a second-person perspective

Currently, internal sensations are assessed by observing behavioral activities either in the form of locomotion or speech or other motor activities. We are not directly examining the internal sensations; in fact, they remain completely unexplored. The depth of this issue can be seen in the following example. Let us imagine an individual with locked-in syndrome (a condition in which a patient is aware of her or his surroundings, but cannot move or verbalize due to complete paralysis of all the voluntary muscles except for the eyes and eyelids) who loses both the eyeballs and eyelids (let us name this hypothetical individual as LISNE (Locked-In Syndrome with No Eyeballs and Eyelids)). Given that an individual with locked-in syndrome is functionally capable of much, even of authoring a book [18], it can be argued that there is a phenomenal limitation in our ability to sense the internal sensations of another nervous system. In the current state of our knowledge, LISNE (for those who are unfamiliar to this individual) or a machine equivalent to LISNE will be completely ignored despite being capable of making internal sensations. Even though functional magnetic resonance imaging (fMRI) signals can be used to identify blood oxygenation level-dependent (BOLD) signals from locations of the brain in response to specific commands, their occurrence after a delay of up to 4 seconds following neuronal activities at the same location [19,20] (normal synaptic delay is only 1 to 2 milliseconds) does not provide information of real-time locations of synaptic activities from which to derive sensory equivalents of internal sensations. This become more confounded due to the fact that the resolving power of fMRI signals does not match with the synaptic or even the neuronal sizes.

### Gating through inter-postsynaptic functional LINKs induces units of internal sensations

**Figure 1:**
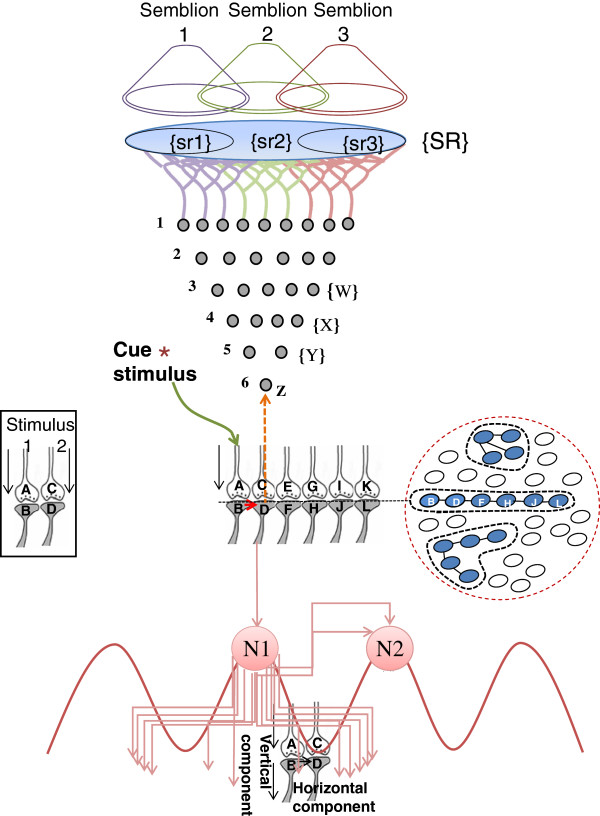
**Cartoon showing lateral entry of activity from a cue stimulus inducing a) formation of semblances, and b) activation of neurons that otherwise would not have activated in the absence of prior associated learning.** When activity from two different sensory receptors passes through their synapses A-B and C-D (in square box) simultaneously, during associative learning, a functional LINK is formed between the postsynapses B and D. After learning, the cue stimulus reaching postsynpse B re-activates (gates through) the inter-postsynaptic functional LINK between the postsynapses B and D and activates postsynapse D, evoking the cellular hallucination (semblance) at the postsynapse D that it is being activated by an action potential reaching its presynapse C, one of the axonal terminals of the neuron Z. The sensory meaning of this hallucination is that the postsynapse D is receiving activity from the neuron Z, which is normally activated by a set of neurons {Y} at its lower neuronal orders that synapse to it. The set of neurons {Y} is normally activated by the activation of the set of neurons {X}, which in turn are activated by the set of neurons {W}. Continuing this extrapolation towards the sensory receptor level identifies a set of sensory receptors {SR}. Subsets of {SR} namely {sr1}, {sr2}, and {sr3} are capable of independently activating the neuron Z. The hypothetical packets of sensory stimuli capable of activating {sr1}, {sr2}, and {sr3} are called semblions 1, 2 and 3 respectively. Activation of the postsynapse D contribute additional EPSP to the neuron N1 that otherwise receives only sub-threshold activation, leading to its activation. Vertical vector for the oscillating neuronal activities is contributed by normal synaptic transmission and horizontal vector by lateral spread of activity through inter-postsynaptic LINKs and recurrent collaterals (shown from neuron N1 to N2). Cross-section through large number of postsynapses is shown within a dotted circle which includes an islet of 6 functionally LINKed postsynapses (B-D-F-H-J-L) along with two additional islets (modified from [23].

From the fact that the stimulation of different locations of the human brain induces hallucinations [21], it can be inferred that when an intermediate node of the system that undergoes oscillatory neuronal activity at certain neuronal orders is stimulated, the system perceives internal sensations as an intrinsic property. Therefore, the lateral entry of activity from the cue stimulus at specific locations within the neuronal pathway at which associative learning-induced changes have taken place should have an operational cellular mechanism for inducing internal sensations at the time of memory retrieval. Such a property is hypothesized to occur through the inter-postsynaptic functional LINKs [22] that are viewed as re-activable, reversible and stabilizable structural features. Re-activation (gating) of the inter-postsynaptic functional LINKs is expected to induce the basic units of internal sensations (semblances), namely semblions (Figure 1). The combinatorial integration of semblions from different neuronal orders is expected to induce different higher brain functions [23]. Continued associative learning events lead to the formation of functional LINKs between the postsynapses that already had established functional LINKs with other postsynapses resulting in the formation of islets of functionally LINKed postsynapses (Figure 1). These islets can be viewed as shared hubs of the wiring.

Oscillatory neuronal activities at different neuronal orders induce sub-threshold activation of different neurons at their higher orders. Re-activation of the functional LINKs during memory retrieval can result in the activation of these sub-threshold activated neurons that are often reported as neuronal activities representing memories from a second-person perspective (for example, neurons N1 and N2 in Figure 1). Oscillating neuronal activity is viewed as a system requirement for semblance formation and can be viewed as being maintained by two key mechanisms: a) Normal synaptic transmission and recurrent connections providing the vertical vector. b) Lateral spread of activity through the inter-postsynaptic functional LINKs providing the horizontal vector. The oscillating neuronal activity results in the activation of a random set of inter-postsynaptic functional LINKs and the resulting net non-specific semblances are expected to be responsible for consciousness [24].

### Technical aspects of replicating the mechanism in physical states

**Figure 2:**
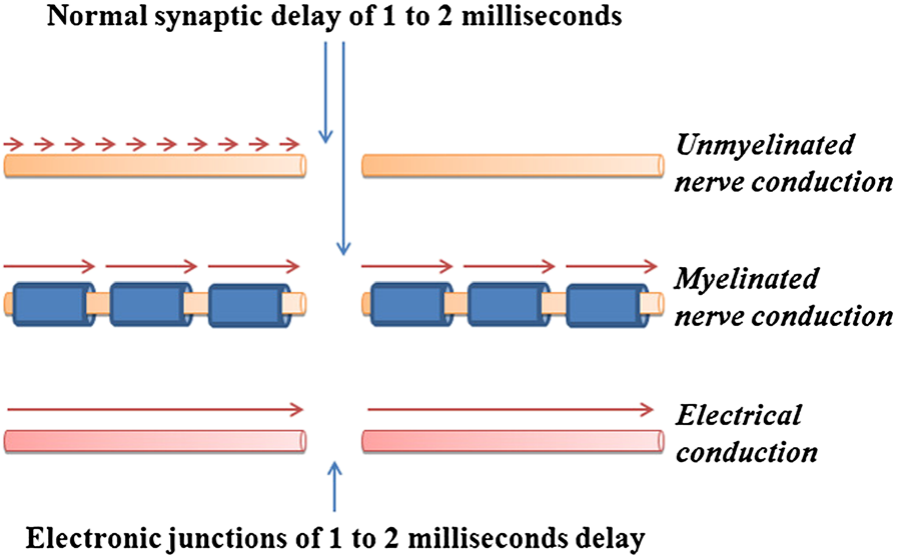
**Different modes of conduction at the inter-synaptic (inter-nodal) area.** The system properties that produce internal sensations are more likely to occur at the level of interactions of its subsystems and unlikely to depend on the details of operation within the subsystems. Due to the continuous interruption of the conduction at the synapses (nodes) with a synaptic delay of 1 to 2 milliseconds, the mechanism of conduction along the inter-synaptic (inter-nodal) length is unlikely to influence the emerging systems properties. **A**. Anterograde (forward) nerve conduction by continuous depolarization and repolarization of membrane potentials. **B**. Myelinated fibers induce large jumps in the above mode of transmission, called saltatory nerve conduction. **C**. Electrical conduction through circuits with an artificially introduced delay of 1 to 2 milliseconds at the junctions (nodes).

**Figure 3:**
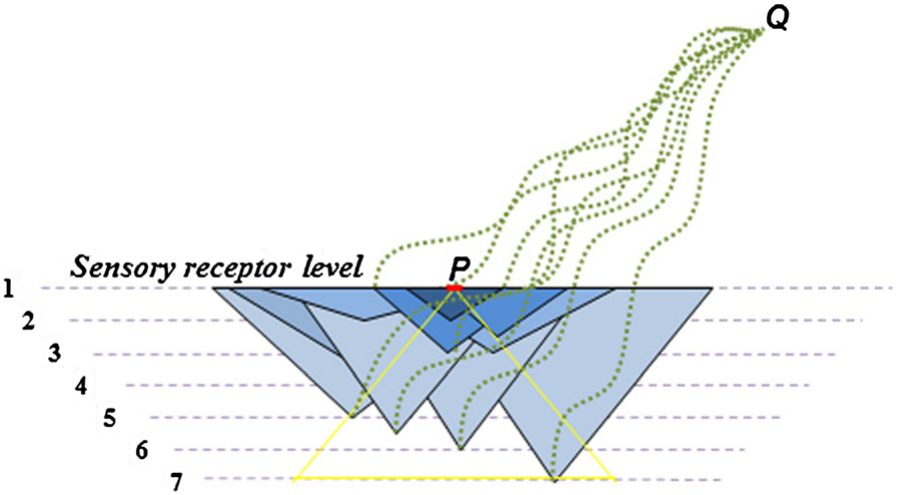
**Cartoon showing overlapping sensory receptors that identify the nature of the induced semblions.** Associative learning of stimulus P with stimulus Q induces inter-postsynaptic functional LINKs at different neuronal orders from 1 to 6 (shown within the yellow triangle). During memory retrieval by the cue stimulus Q, large number of inter-postsynaptic functional LINKs within the yellow triangle area gets re-activated and semblions at their corresponding postsynapses are induced (represented by blue-colored triangles). The maximum overlap of sensory receptors (shown as a red line at the level of sensory receptors) represents the overlap or integral of the semblions, resulting in internal sensation.

a) *Nerve conduction in terms of electron flow:* Nerve conduction takes place by the spread of depolarization along the neuronal membranes [25] and is interrupted at the junctions between the neurons with a synaptic delay of 1 to 2 milliseconds. For an emergent systems property of a system with an inter-nodal (synaptic) delay of 1 to 2 milliseconds and which is required to have oscillatory neuronal activity at certain neuronal orders, the mode of nerve conduction at the inter-nodal areas may not be a determining factor for the emergent property of internal sensations (Figure 2). This may enable replicating the mechanism using direct current electronic circuits by introducing synaptic delay at the uni-directionally operating diodes that function as synapses.

b) *Spatial amplification of signals*: Activation of a synapse induces excitatory postsynaptic potential (EPSP) at the postsynaptic terminal that propagates towards the cell body. Summation of nearly 40 EPSPs arriving from any 40 synapses (out of an average 2.4-8x10^4^ dendritic spines [26] can trigger an action potential at the axon hillock (an area immediately after the cell body, at the beginning of the main axon) that can then spread to all the axonal (presynaptic) terminals of the neuron (for theoretical purposes, an average number of 2.4-8x10^4^). This indicates that the electronic equivalent of the axon hillock should have a function of a direct current step-up transformer. Since the output of the system is not an amplified input sensory signal, we may treat the sensory inputs as a source of electricity (similar to photo-electricity).

c) *Gradual decay of signals:* It is known that excitatory postsynaptic potentials (EPSPs) from apical synapses of cortical pyramidal cells do not reach the cell body [27] and will not contribute to the summation of EPSPs at the axon hillock for action potential generation. Cables of dendrites should have a gradient of diameters which decrease as the distance from the cell body increases. The expected loss of energy by heat dissipation necessitates the provision of a heat sink at the dendritic area of the system.

d) *Introducing functional LINKs in an electronic circuit model*: Inter-postsynaptic functional LINKs may be introduced using an AND logic gate that function when both postsynapses are activated simultaneously. If this can lead to the activation of a silicon-controlled rectifier (SCR) that leads to the opening of an OR gate between these postsynapses, then at a later time-point the activation of either one of the postsynapses may be able to open this OR gate (similar to the re-activation of an inter-postsynaptic functional LINK). Artificial systems with these functions may be examined for semblance formation.

e) *Maintaining appropriate oscillating neuronal activities:* The formation of semblances is seen as an emergent property of a system with oscillatory neuronal activity. Fine-tuning of systems properties to match the formed internal sensations of retrieved memories of an item to that of the actual properties of the item used in associative learning is the final step of the process. The knowledge that changes in the frequency of oscillations during sleep [28] and anesthesia [29] changes the level of consciousness and consequently the ability to form internal sensations can be utilized while fine-tuning the frequency and amplitude of the oscillations.

f) *Overlapping semblances determine the identity of the internal sensations*: Inputs (EPSPs) summated at the axon hillock from nearly any 40 postsynaptic terminals give rise to an action potential, which in turn activates nearly 2.4-8x10^4^ axonal (presynaptic) terminals and their corresponding postsynaptic terminals in the next layer. Therefore, an action potential generated is not input-specific (not specific to the set of EPSPs that induces action potential). This information can be used while extrapolating for the semblions from a postsynapse activated through inter-postsynaptic functional LINK by lateral entry of activity from the cue stimulus (Figure 1). The interpretation that an action potential produced would have been elicited by the spatial summation of any set of 40 dendritic spines (postsynapses) of the neuron indicates large number of potential semblions as perceived units of internal sensations. This necessitates retrograde examination from a re-activated postsynapse to include all the possible semblions for that particular neuron for extrapolating the net semblance (Figure 1). The net overlapped sensory dimension of semblions from different neuronal orders is likely to determine the conformation of the net semblance for the retrieved memory (Figure 3).

g) *Recapitulating ontogeny during AI development:* It is important that the construction of the artificial systems should recapitulate the developmental stages of the nervous system. In this regard, discontinuity of the tracings in the electroencephalogram (EEG) among very premature infants [30] suggests a lack of lateral spread of activity during this stage of development. They eventually develop continuous EEG tracings, possibly due to the lateral spreading of neuronal activity through the formation of inter-postsynaptic functional LINKs and the sprouting collaterals.

h) *Obtaining logic output to identify internal sensations:* What methods will allow us to obtain a read-out for the nature of semblions? At the behavioral level, monkeys were conditioned to respond to electrical stimulation delivered to different layers within the visual cortex V1 [31]. By inducing a pressure phosphene (very slight pressure over the lateral aspect of the eyeball after closing the eyes stimulates the horizontal cell layer in the retina and induces an internal sensation of light in complete darkness) of a similar nature, experimenters were able to prompt monkeys to respond with learned motor actions. It was found that the excitabilities of V1 neuronal regions are similar to that produced during stimulation-induced phosphenes in the human V1 region [32]. Can we implement a strategy similar to this in machines, with the assumption that the units of internal sensations are being induced simultaneously with the firing of otherwise sub-threshold activated neurons (Figure 1) when the system receives certain sensory inputs (pressure to induce phosphenes or cue stimulus for memory retrieval)? The correlation between the concurrent activation of these neurons with the nature of expected semblances can be explored from the structural architecture of the system.

This letter has highlighted the technical aspects of engineering a feasible system in physical states based on the hypothesis of the formation of semblances, both to understand the intrinsic science behind the formation of internal sensations and to replicate the mechanism in artificial systems. The existence of diverse types of nervous systems in the animal kingdom organized in a wide range of sequences of the same expected operational units lends hope to the goal successfully transferring the mechanism to physical systems.

## Abbreviations

BOLD: Blood Oxygenation Level Dependent; EEG: Electro-encephalogram; fMRI: Functional Magnetic Resonance Imaging; LISNE: Locked In Syndrome with No Eyeballs and Eyelids; TBI: Traumatic Brain Injury.

## Competing interests

The authors declare that they have no competing interests.
